# The Role of Childhood Adversity and Social Drivers of Health in Subjective Cognitive Decline

**DOI:** 10.5888/pcd22.250116

**Published:** 2025-07-17

**Authors:** Aishwarya Joshi, Jungwon Yeo

**Affiliations:** 1 University of Central Florida, Orlando; 2 University of Maryland, Baltimore County, Baltimore

## Abstract

**Introduction:**

Cognitive health is influenced by a complex interplay of factors throughout the lifespan. Identifying childhood adversities and social needs can be important in mitigating subjective cognitive decline and promoting healthy aging. This study analyzes the role of social drivers of health on adverse childhood experiences and subjective cognitive decline.

**Methods:**

We conducted structural equation modeling on data from the 2023 Behavioral Risk Factor Surveillance System to investigate the association among adverse childhood experiences, social drivers of health, and subjective cognitive decline in a sample of adults aged 45 years or older (n = 35,754).

**Results:**

In our study sample, 17.7% reported experiencing subjective cognitive decline within the past 12 months. Adverse childhood experiences were significantly associated with greater subjective cognitive decline (β = 0.136, *P* < .001). Adverse childhood experiences were negatively associated with both social drivers of health, perceived social support (β = −0.517, *P* < .001), and socioeconomic stability (β = −0.022, *P* = .047). However, greater perceived social support (β = −0.260, *P* < .001) and socioeconomic stability (β = −0.086, *P* < .001) reduced the effects of adverse childhood experiences on subjective cognitive decline.

**Conclusion:**

Adverse childhood experiences were significantly associated with subjective cognitive decline; however, this association was attenuated when mediated by perceived social support and socioeconomic stability. These findings can inform public health providers and policymakers to implement targeted interventions, such as promoting resilience, reinforcing nurturing parenting styles, strengthening community networks, and integrating behavioral health into primary care settings.

**Summary**

**What is already known on this topic?**

Cognitive decline is complex and multifaceted and is influenced by various social drivers of health and early-life chronic stress.

**What is added by this report?**

This study reinforces evidence for the association between adverse childhood experiences and subjective cognitive decline and introduces social drivers of health — perceived social support and socioeconomic stability — as key mediators in this relationship. Social drivers of health significantly attenuated the association between adverse childhood experiences and subjective cognitive decline.

**What are the implications for public health practice?**

Public health practitioners can use these findings to identify potential intervention points, allowing for more targeted strategies to improve cognitive outcomes among adults.

## Introduction

The US is experiencing a substantial demographic shift, with the population aged 45 years or older projected to increase from 42.1% in 2022 to 48.5% in 2050 (1). As this population grows, the number of people affected by cognitive impairment is expected to rise (2). Approximately 11% of people aged 45 years or older report experiencing subjective cognitive decline (SCD), characterized by self-perceived worsening of memory or more frequent confusion and memory loss (3). Preventing SCD is crucial, because it can be a precursor to dementia and Alzheimer disease. By 2050, the prevalence of Alzheimer disease is projected to affect 82 million Americans, with associated costs reaching $1 trillion (4). Prioritizing proactive measures to maintain cognitive health can promote healthy aging, improve overall well-being, and reduce the burden on health care systems and society.

The life course theory suggests that social, cultural, economic, and environmental experiences accumulate over time, shaping health outcomes in older age (5,6). Early-life experiences, including relationships with parents and other adults, household routines, school engagement, and neighborhood cohesion, are significantly associated with long-term health outcomes (7,8). Positive childhood experiences emerging from family stability and positive role models foster emotional security, strengthen coping skills, and support healthy development into adulthood (7,8). Conversely, adverse childhood experiences (ACEs) involving early-life stress and family adversity can initiate delayed physiological and psychological processes that undermine long-term health (7,8). According to the ACE Pyramid developed by the Centers for Disease Control and Prevention (CDC) and Kaiser Permanente, social and emotional impairment and the adoption of health-risk behaviors are key mechanisms through which early childhood adversity contributes to poor health outcomes in adulthood (9–11).

Previous studies demonstrated a connection between ACEs and cognitive deterioration in later life, including difficulties with memory tasks, attention, and daily functioning (11–20). Risk factors such as health-risk behaviors and inflammation are associated with this relationship (10,15,20). However, the underlying mechanisms mediating this relationship are poorly understood. This study contributes to the current understanding of the ACE–SCD relationship by highlighting the mediating role of social drivers of health, including perceived social support and socioeconomic stability. Our findings can help identify potential points of intervention, allowing for targeted and effective strategies to improve cognitive outcomes among adults.

## Methods

We used data from the 2023 Behavioral Risk Factor Surveillance System (BRFSS) survey for analysis. The BRFSS, established by CDC, is an annual telephone survey that collects health information from noninstitutionalized US residents about health-related risk behaviors, chronic health conditions, and use of preventive services (21). The survey is conducted among adults aged 18 years or older in 50 states, Washington, DC, the US Virgin Islands, Puerto Rico, and Guam. Kentucky and Pennsylvania were excluded from the 2023 public dataset because they did not collect sufficient data to meet the minimum inclusion criteria (21). BRFSS survey data are weighted to reflect known proportions of age, sex, ethnicity, geographic regions within states, marital status, education level, home ownership, and type of telephone ownership. Total sample size for the 2023 survey was 433,323.

The BRFSS dataset has a set of core and optional modules. While core modules are administered nationwide, optional modules are selected by states based on data needs and research priorities. To address our research questions, we used a subsample from the 12 states (Delaware, Florida, Georgia, Maryland, Missouri, Nevada, New Jersey, Ohio, Oregon, Rhode Island, Tennessee, and Virginia) that administered both ACE and SCD optional modules in 2023. The SCD module is administered exclusively to adults aged 45 or older (3,14). In an initial sample of 40,338 participants that met inclusion criteria, 4,584 participants had missing responses or responded “don’t know/not sure” on SCD and ACEs questions. Our final sample consisted of 35,754 participants aged 45 years or older in 12 states.

### Measures

#### Endogenous (dependent) variable

The endogenous variable was SCD, self-reported cognitive decline measured as a binary outcome based on the question, “During the past 12 months, have you experienced confusion or memory loss that is happening more often or is getting worse?” Responses were coded as yes or no.

#### Exogenous (independent) variable

The exogenous variable was ACEs. Guided by prior research (14,19), we created indices to measure ACEs using 11 validated questions from the dataset. To provide a nuanced and interpretable understanding of ACEs, we grouped these questions into 3 indices: 1) unstable household (6 questions) consisting of questions on household drug use, alcohol use, mental illness, domestic violence, parental separation/divorce, and incarcerated family member; 2) physical or emotional abuse (2 questions); and 3) sexual abuse (3 questions) (14,22). Each response was dichotomized as yes or no. For questions about witnessing domestic violence, physical abuse, and sexual abuse, responses of “once” or “more than once” were coded as yes, and responses of “never” were coded as no. We then summed all affirmative responses within their respective indices to calculate cumulative ACE scores. If participants experienced ACEs across multiple categories, they received 1 point per item in each relevant domain. We created a latent variable, ACEs, by using these 3 observed variables. The operationalization of these variables is provided (Appendix).

#### Mediating variables

From the BRFSS module on social determinants of health, we included 4 variables: education, annual household income, perceived emotional support, and perceived loneliness. We grouped these variables into 2 latent constructs representing social drivers of health — socioeconomic stability (education and annual household income) and perceived social support (perceived emotional support and perceived loneliness). We examined these constructs as potential mediators in the proposed pathway between ACEs and SCD.

We measured socioeconomic stability by the observed variables of education and annual household income. Education was measured from 1 to 4 in the following categories: did not graduate from high school, graduated from high school, attended college or technical school, graduated from college or technical school. Annual household income was measured from 1 to 7 in the following categories: <$15,000, $15,000 to <$25,000, $25,000 to <$35,000, $35,000 to <$50,000, $50,000 to <$100,000, $100,000 to <$200,000, and ≥$200,000.

Perceived social support was measured by 2 observed variables, perceived emotional support and perceived loneliness (12,17,23). As in previous studies (24,25), we interpreted responses from study participants as perceived social support because the survey did not assess actual social support. Perceived social support refers to a person’s belief that support will be available when needed (26). Perceived emotional support was measured by the question, “How often do you get the social and emotional support you need?” Survey participants responded on a 5-item Likert scale, which we converted into a binary variable. We assigned “always,” “usually,” and “sometimes” as 1 and “rarely” and “never” as 0. Perceived loneliness was measured by the question, “How often do you feel lonely?” The scale was reverse coded by assigning “rarely” and “never” as 1 and “always,” “usually,” and “sometimes” as 0.

#### Control variables

This study controlled for sex (male vs female), race and ethnicity (non-Hispanic White vs Other [comprising Hispanic, non-Hispanic American Indian or Alaska Native, non-Hispanic Asian, non-Hispanic Black, non-Hispanic Native Hawaiian or Other Pacific Islander, non-Hispanic Other race, and non-Hispanic multiracial]), marital status (married or living with significant other vs single), and self-reported health status (5 categories: excellent, very good, good, fair, and poor) (10,12–14).

### Statistical analysis

We used the weighted BRFSS dataset to generate descriptive statistics. Next, we used a confirmatory factor analysis (CFA) to determine whether the 11 ACE items loaded onto the 3 indices (unstable household, physical or emotional abuse, and sexual abuse) (22). We also used CFA to assess the fit of 2 latent constructs related to social drivers of health — perceived social support and socioeconomic stability. In line with standard practices, we used a factor loading threshold of 0.40 or higher to retain the variables within their respective latent constructs (11,27). Once we confirmed the measurement model, we used structural equation modeling (SEM) to assess the mediating effects of perceived social support and socioeconomic stability on the relationships between ACEs and SCD. Because the analyses included categorical variables, models were estimated with weighted least squares mean and variance. We addressed missing data by using a pairwise present approach to retain the maximum available information (23). Following published guidelines, we assessed CFA and SEM fits by reviewing the Comparative Fit Index (CFI) and Tucker-Lewis Index (TLI). We considered values greater than 0.90 as indicative of acceptable model fit to account for the complexity and measurement variability in large-scale population-based datasets (27,28). We conducted all analyses in R version 2024 (R Development Core Team) using the lavaan package version 0.6-19 (Yves Rosseel). The BRFSS survey consists of de-identified, publicly accessible data that do not qualify as research involving human subjects and, therefore, did not require institutional review board approval.

## Results

Of the 35,754 participants, 54.1% were female, 46.0% were aged 65 years or older, 68.6% were non-Hispanic White, 64.0% were married or living with a significant other, 32.8% indicated good health status, 28.9% had annual household income from $50,000 to less than $100,000, 34.1% graduated from college or technical school, and more than 90% always, sometimes, or usually had perceived emotional support and rarely or never felt lonely (Table 1). Overall, 17.7% of respondents experienced SCD, 46.3% experienced an unstable household, 39.1% experienced physical or emotional abuse, and 12.5% experienced sexual abuse.

**Table 1:** Characteristics of Participants (N = 35,754) in Optional Modules (Adverse Childhood Experiences and Subjective Cognitive Decline) of the Behavioral Risk Factor Surveillance System Survey, 12 States, 2023^a^

Characteristic	No. (weighted %)^b^
**Subjective cognitive decline**	
Yes	6,113 (17.7)
No	29,641 (82.3)
**Age group, y**	
45–54	6,411 (24.9)
55–64	8,959 (29.2)
≥65	20,384 (46.0)
**Sex**	
Male	9,511 (45.9)
Female	12,639 (54.1)
**Race and ethnicity**	
Non-Hispanic White	28,574 (68.6)
Other^c^	6,541 (31.4)
**Marital status**	
Married or living with a significant other	20,773 (64.0)
Single	14,765 (36.0)
**General health status**	
Excellent	4,644 (13.3)
Very good	11,805 (32.0)
Good	11,659 (32.8)
Fair	5,495 (16.0)
Poor	2,066 (5.9)
**ACEs**	
Unstable household, no. of ACEs^d^	
0	19,754 (53.7)
1	8,299 (23.8)
2	3,988 (11.4)
3	2,083 (6.0)
4	1,014 (3.1)
5	439 (1.5)
6	177 (0.6)
Physical and emotional abuse, no. of ACEs^e^	
0	22,084 (60.9)
1	8,499 (24.3)
2	5,171 (14.9)
Sexual abuse, no. of ACEs^f^	
0	31,246 (87.5)
1	1,878 (4.8)
2	1,454 (4.2)
3	1,176 (3.5)
**Socioeconomic stability**	
Annual household income, $	
<15,000	1,477 (5.3)
15,000 to <25,000	2,795 (9.7)
25,000 to <35,000	3,287 (10.6)
35,000 to <50,000	3,865 (13.2)
50,000 to <100,000	9,155 (28.9)
100,000 to <200,000	6,456 (23.3)
≥200,000	2,301 (9.0)
Education	
Did not graduate from high school	1,861 (9.0)
Graduated from high school	8,387 (25.9)
Attended college or technical school	9,312 (31.0)
Graduated from college or technical school	16,098 (34.1)
**Perceived social support**	
Perceived emotional support^g^	
Always/sometimes/usually	21,469 (94.0)
Rarely/never	1,165 (6.0)
Perceived loneliness^h^	
Rarely/never	21,633 (94.8)
Always/sometimes/usually	1,126 (5.2)

Abbreviation: ACE, adverse childhood experience.

a The 12 states are Delaware, Florida, Georgia, Maryland, Missouri, Nevada, New Jersey, Ohio, Oregon, Rhode Island, Tennessee, and Virginia.

b Values calculated by using the appropriate weighting system. Percentages may not sum to 100 because of rounding.

c Comprises Hispanic, non-Hispanic American Indian or Alaska Native, non-Hispanic Asian, non-Hispanic Black, non-Hispanic Native Hawaiian or Other Pacific Islander, non-Hispanic Other race, and non-Hispanic multiracial.

d Number of times survey respondent answered yes to the 6 questions on ACEs.

e Number of times survey respondent answered yes to the 2 questions on ACEs.

f Number of times survey respondent answered yes to the 3 questions on ACEs.

g Survey question was “How often do you get the social and emotional support you need?”

h Survey question was “How often do you feel lonely?”

The CFA for each latent construct — ACE, socioeconomic stability, and perceived social support — demonstrated an appropriate factor structure with strong model fit (CFI = 0.995; TLI = 0.991). Factor loadings ranged from 0.573 to 0.948, reflecting satisfactory contributions to their respective constructs: unstable household, physical or emotional abuse, and sexual abuse for ACE; education and annual household income for socioeconomic stability; and perceived emotional support and perceived loneliness for perceived social support (Figure).

**Figure:**
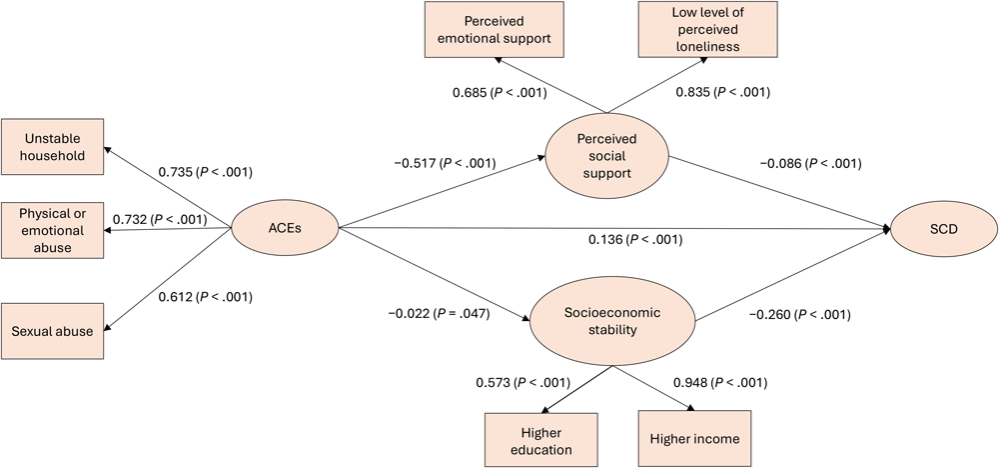
Structural equation modeling pathway between adverse childhood experiences (unstable household, physical or emotional abuse, and sexual abuse), subjective cognitive decline, and social drivers of health (perceived social support and socioeconomic stability) among adults aged ≥45 years, 12 states, Behavioral Risk Factor Surveillance System Survey, 2023. The model has a satisfactory fit (Comparative Fit Index = 0.901, Tucker-Lewis Index = 0.923). Abbreviations: ACE, adverse childhood experience; SCD, subjective cognitive decline.

**Table:** 

Pathway	Estimate (*P* value)
ACE to SCD	0.136 (<.001)
ACE to perceived social support	−0.517 (<.001)
ACE to socioeconomic stability	−0.022 (.047)
ACE to unstable household	0.735 (<.001)
ACE to physical or emotional abuse	0.732 (<.001)
ACE to sexual abuse	0.612 (<.001)
Socioeconomic stability to SCD	−0.260 (<.001)
Socioeconomic stability to higher education	0.573 (<.001)
Socioeconomic stability to higher income	0.948 (<.001)
Perceived social support to SCD	−0.086 (<.001)
Perceived social support to perceived emotional support	0.685 (<.001)
Perceived social support to low level of perceived loneliness	0.835 (<.001)

Similarly, SEM showed an acceptable model fit for the SCD construct (CFI = 0.901; TLI = 0.923). ACEs accounted for 1.7% of the variance in SCD (*R* ^2^ = 0.017), while ACEs, perceived social support, and socioeconomic stability collectively accounted for 23.4% of the variance in SCD (*R* ^2^ = 0.234). ACEs were associated with a higher risk of SCD (β = 0.136, *P* < .001) (Figure, Table 2). ACEs were negatively associated with both social drivers of health, perceived social support (β = −0.517, *P* < .001) and socioeconomic stability (β = −0.022, *P* = .047). Finally, perceived social support (β = −0.086, *P* < .001) and socioeconomic stability (β = −0.260, *P* < .001) reduced the effects of ACEs on SCD.

**Table 2:** Structural Equation Model Estimates Showing Strength and Direction of the Relationship Among ACEs, Subjective Cognitive Decline, and Social Drivers of Health Among Adults Aged ≥45 Years, Behavioral Risk Factor Surveillance System Survey, 2023^a^

Pathway	Estimate, β^b^ (SE)	*P* value
ACE to subjective cognitive decline	0.136 (0.022)	<.001
ACE to perceived social support	−0.517 (0.030)	<.001
ACE to socioeconomic stability	−0.022 (0.011)	.047
Socioeconomic stability to subjective cognitive decline	−0.260 (0.026)	<.001
Perceived social support to subjective cognitive decline	−0.086 (0.013)	<.001

Abbreviations: ACE, adverse childhood experience.

a ACEs are unstable household, physical or emotional abuse, and sexual abuse; social drivers of health are perceived social support and socioeconomic stability.

b Adjusted for control variables, including race, sex, marital status, and general health status.

## Discussion

This study examined the relationship between ACEs and SCD, with a focus on social drivers of health, including perceived social support and socioeconomic stability as potential mediators. The findings add to existing research by introducing latent constructs of social drivers of health, offering new insights into how they may mediate the effect of early adversity on cognitive health. Consistent with previous research, our study indicated that ACEs, including unstable households, physical or emotional abuse, and sexual abuse, significantly affect SCD (11–20). Brown et al found that sexual, physical/psychological, and environmental ACEs were significantly associated with SCD among people aged 45 years or older (14). Children exposed to adversity are at heightened risk of developing toxic stress, which disrupts both immune and neurological functioning (11,14,15,29). This stress may contribute to the dysregulation of the hypothalamic–pituitary–adrenal axis and alter brain regions, including the hippocampus, prefrontal cortex, and amygdala, which are essential for memory formation and retention (15,18,29). Additionally, ACEs may contribute to cognitive decline indirectly through depressive symptoms and chronic inflammation, reflected by elevated levels of interleukin-6 and C-reactive protein in adulthood (15,20). These neurobiological effects of childhood adversity may help explain the observed association between ACEs and an increased risk of SCD in our study.

Our study indicated that perceived social support and socioeconomic stability significantly mediated the association between ACE and SCD, suggesting that social drivers of health can play a crucial role in offsetting the long-term negative effects of early adversity. Prior research showed that perceived social support serves as a critical buffer against the behavioral and health effects of ACEs (12,18,19,26,29). Early life trauma can impair emotional regulation and social functioning, making it more difficult to manage stress and increasing the likelihood of maladaptive coping behaviors such as smoking, substance use, and poor dietary habits (15,17,18). Engaging in social activities and maintaining strong interpersonal connections can serve as protective mechanisms to counteract the psychological and physiological effects of this early trauma (17,29,30). People who have access to strong social networks and use adaptive coping strategies have greater resilience and report fewer functional limitations than people without such networks (18,26). Lin et al suggested that increased social interaction may mitigate the negative effects of ACEs by promoting mental stimulation, reducing psychological distress, and encouraging healthy behaviors (17). Hence, fostering supportive environments throughout the life course is essential to mediate the effects of ACEs and promote healthier cognitive aging.

Poverty exacerbates the incidence of ACEs, leading to financial instability that continues into adulthood (13). The link between income inequality and health outcomes may be influenced by broader structural factors, such as the unequal distribution of public goods and services (31). People from low-income backgrounds often have limited access to resources such as health services and high-quality education (28,31). Preventive care, such as regular cognitive assessments and management of chronic conditions, plays a critical role in delaying or mitigating age-related cognitive deterioration (32). However, people with lower socioeconomic status face substantial barriers, including cost, lack of health insurance, and transportation challenges, that hinder their ability to obtain early screening and timely interventions for cognitive decline (32). Additionally, limited education reduces engagement in mentally stimulating activities, including skill acquisition, routine development, and social interaction, contributing to cognitive resilience (12,16,31). Higher education also increases employment opportunities, fostering financial stability and enabling early detection and treatment of cognitive decline (31). This cyclical relationship perpetuates disparities: restricted educational attainment limits job prospects and economic mobility, while financial instability constrains access to education and cognitively enriching opportunities. These findings underscore the importance of economic stability and higher education as a potential pathway through which early-life adversities may influence long-term cognitive outcomes.

### Strategies to mitigate the risk of cognitive decline

Our study demonstrated a significant negative association between ACEs and social drivers of health. This finding supports existing research indicating that early life adversity can disrupt long-term social functioning and economic opportunities (12,16). We propose strategies based on this evidence to mitigate these effects and reduce the risk of cognitive decline in older adults. Identifying and addressing childhood trauma is an important step in promoting healthy aging and preventing cognitive decline. Trauma-informed techniques that recognize the lasting effect of ACEs can support people at risk of developing SCD (18,19). Health care professionals should be given trauma-informed care training to help create individualized treatment programs that promote resilience and prevent cognitive decline. Several programs, such as Early Head Start, SafeCare, and The Incredible Years, are available to professionals to help them understand, recognize, and prevent ACEs (33).

Public health efforts to prevent childhood toxic stress must promote safe, stable, and nurturing relationships through a tiered approach. Primary prevention includes universal strategies to reduce stressors such as abuse, poverty, and social isolation (29). Secondary prevention supports children at risk of ACEs through targeted interventions such as HealthySteps and screenings for food insecurity or postpartum depression (29). Tertiary prevention supports symptomatic children with evidence-based therapies such as the Attachment and Biobehavioral Catch-up program, parent–child interaction therapy, and child–parent psychotherapy (29). Family-centered pediatric medical homes can implement this tiered approach by addressing structural barriers, incorporating training in therapeutic methods, and fostering cross-sector collaboration with education, justice, and social service systems to build strong medical neighborhoods (29).

To create an environment where children can thrive, pediatric care systems should prioritize teaching and reinforcing nurturing parenting styles characterized by warmth, consistency, and positive discipline (29). These nurturing styles are crucial for building children’s core life skills like emotional regulation and executive function. Providers can integrate evidence-based supports such as the Reach Out and Read program and the Video Interaction Project to coach caregivers in responsive, structured, and emotionally supportive parenting (29). Public health strategies should strengthen community networks through trusted institutions such as schools, recreation centers, and civic organizations and expand access to supportive environments such as parks and green spaces. These measures can enhance the sense of belonging, reduce isolation, and promote resilience for people experiencing ACEs (29,34). For adults experiencing SCD, expanding access to age-friendly social activities, increasing public awareness about the importance of social engagement, and using appropriate technology-based solutions to support memory can foster internal motivation and promote active participation (35).

Lastly, behavioral health should be integrated into the primary care setting; this integration could provide a comprehensive approach to overcome the complex effects of ACEs (18). Primary care clinicians can work collaboratively with behavioral health specialists to provide coordinated care and treatment plans to mitigate the negative effects of ACEs on cognitive function.

### Limitations

The study has several limitations. First, BRFSS collects self-reported data that may be subject to recall or social desirability bias, leading to inaccurate reporting. Second, BRFSS data do not include participants who are homeless or reside in long-term care facilities, limiting our study sample. These populations may experience a higher prevalence of ACEs and poor cognitive and social outcomes, limiting the external validity and generalizability of our findings. Third, the study is subject to limitations in construct validity. The BRFSS data lack the clinically measured variables necessary to examine important factors of cognitive decline. Additionally, the construct of perceived social support is complex, and our reliance on just 2 self-reported items may not fully capture its multidimensionality. Similarly, we constructed race and ethnicity as a binary variable, which may have obscured the heterogeneity of the category. Fourth, the study’s cross-sectional design prevents any causal inference between ACEs and SCD. Although we used SEM to control for potential confounders, residual confounding may still exist and affect the observed associations. Additionally, we did not conduct subgroup analyses (eg, by race or sex), nor did we perform sensitivity or robustness checks using alternative model structures, limiting the ability to assess consistency across different populations. Lastly, the study did not adjust for other health, behavioral, and chronic disease factors contributing to SCD. Future studies must adjust for these control variables and use a longitudinal approach to shed additional light on how ACEs and cognitive function interact over time. Future studies should also assess the effect of the number of ACEs on SCD within the context of social drivers of health to evaluate a potential dose–response relationship.

### Conclusion

Cognitive decline is complex and multifaceted, influenced by various social drivers of health and ACEs. Our study showed that the social drivers of health can significantly mediate the association between ACEs and SCD. Given the challenges of preventing dementia, public health strategies can prioritize enhancing perceived social support and socioeconomic stability from an early age, particularly for people with ACEs. These strategies provide collaboration opportunities between social workers, pediatricians, and geriatricians to better understand the early life experiences of adults. Public health providers and policymakers should consider implementing interventions focused on promoting resilience, reinforcing nurturing parenting styles, strengthening community networks, and integrating behavioral health into primary care settings.

## Appendix Operationalization of Variable Measures, Study on Adverse Childhood Experiences and Subjective Cognitive Decline, Behavioral Risk Factor Surveillance System, 12 States, 2023

**Table:**

Characteristic	Latent variable	Observed variable	Measure	Question
Exogenous variable	Not applicable	Subjective cognitive decline	Binary	During the past 12 months, have you experienced confusion or memory loss that is happening more often or is getting worse?
Endogenous variable	Adverse childhood experiences	Unstable household	Ordinal (0 to 6)	Did you live with anyone who was depressed, mentally ill, or suicidal?
				Did you live with anyone who was a problem drinker or an alcoholic?
				Did you live with anyone who used illegal street drugs or who abused prescription medications?
				Did you live with anyone who served time or was sentenced to serve time in a prison, jail, or other correctional facility?
				Were your parents separated or divorced?
				How often did your parents or adults in your home ever slap, hit, kick, punch, or beat each other up?
		Physical or emotional abuse	Ordinal (0 to 2)	Not including spanking (before age 18), how often did a parent or adult in your home ever hit, beat, kick, or physically hurt you in any way?
				How often did a parent or adult in your home ever swear at you, insult you, or put you down?
		Sexual abuse	Ordinal (0 to 3)	How often did anyone at least 5 years older than you or an adult ever touch you sexually?
				How often did anyone at least 5 years older than you or an adult, try to make you touch them sexually?
				How often did anyone at least 5 years older than you or an adult, force you to have sex?
Mediating variable	Perceived social support	Perceived emotional support	Binary	How often do you get the social and emotional support you need?
		Perceived loneliness	Binary	How often do you feel lonely?
	Socioeconomic stability	Income	Ordinal (1 to 7)	Annual household income level
		Education	Ordinal (1 to 4)	Level of education completed
Control variable	—	Race	Binary	Computed race: non-Hispanic White vs other
		Sex	Binary	Computed sex: male vs female
		Marital status	Binary	Computed marital status: married or living with significant other vs single
		General health status	Ordinal (1 to 5)	Would you say that in general your health is excellent, very good, good, fair, or poor?
